# TheraPearl Eye Mask and Blephasteam for the treatment of meibomian gland dysfunction: a randomized, comparative clinical trial

**DOI:** 10.1038/s41598-021-01899-8

**Published:** 2021-11-17

**Authors:** Jonatan Olafsson, Xiaoran Lai, Erlend Christoffer Sommer Landsend, Snorri Olafsson, Eric Parissi, Øygunn A. Utheim, Sten Raeder, Reza A. Badian, Neil Lagali, Darlene A. Dartt, Tor P. Utheim

**Affiliations:** 1 Institute of Clinical Medicine, Faculty of Medicine, University of Oslo, Klaus Torgårds vei 3, 0372 Oslo, Norway; 2 Department of Medical Biochemistry, Oslo University Hospital, Nydalen, P.O. Box 4950, 0424 Oslo, Norway; 3 The Norwegian Dry Eye Clinic, Ole Vigs gate 32E, 0366 Oslo, Norway; 4 Department of Biostatistics, Institute of Basic Medical Sciences, University of Oslo, Blindern, P.O. Box 1122, 0317 Oslo, Norway; 5 Department of Ophthalmology, Oslo University Hospital, Nydalen, P.O. Box 4950, 0424 Oslo, Norway; 6 Department of Gastroenterology, Innlandet Hospital Trust, Kyrre Grepps gate 11, 2816 Gjøvik, Norway; 7 Loma Linda University Medical Center, 11234 Anderson St., Loma Linda, CA 92354 USA; 8 Lavista Optometry, Dampsagveien 4, 2000 Lillestrom, Norway; 9 Department of Optometry, Radiography and Lighting, National Centre for Optics, Vision and Eye Care, Faculty of Health and Social Sciences, University of Southeast Norway, P.O. Box 235, 3603 Kongsberg, Norway; 10 Division of Ophthalmology, Department of Biomedical and Clinical Sciences, Linköping University, 581 83 Linköping, Sweden; 11 Department of Ophthalmology, Sørlandet Hospital Arendal, Sykehusveien 1, 4838 Arendal, Norway; 12 Department of Ophthalmology, Schepens Eye Research Institute/Massachusetts Eye and Ear, Harvard Medical School, 20 Staniford St., Boston, MA 02114 USA; 13 Department of Ophthalmology, Stavanger University Hospital, P.O. Box 8100, 4068 Stavanger, Norway

**Keywords:** Eyelid diseases, Corneal diseases

## Abstract

Meibomian gland dysfunction (MGD) is the most common cause of dry eye disease (DED). In this study, we aimed to compare the effects of eyelid warming treatment using either TheraPearl Eye Mask (Bausch & Lomb Inc., New York, USA) or Blephasteam (Spectrum Thea Pharmaceuticals LTD, Macclesfield, UK) in a Norwegian population with mild to moderate MGD-related DED. An open label, randomized comparative trial with seventy patients (49 females, 21 males; mean age 53.6 years). Patients were randomly assigned to treatment with Blephasteam (n = 37) or TheraPearl (n = 33). All received a hyaluronic acid based artificial tear substitute (Hylo-Comod, Ursapharm, Saarbrücken, Germany). Patients were examined at baseline, and at three and six months initiation of treatment. Treatment efficacy was primarily evaluated by fluorescein breakup time (FBUT) and Ocular Surface Disease Index (OSDI) scores. Other outcome measures included ocular surface staining (OSS), Schirmer’s test, and meibomian quality and expressibility. Baseline parameter values did not differ between the groups. After six months of treatment, Blephasteam improved FBUT by 3.9 s (p < 0.01) and OSDI by 13.7 (p < 0.01), TheraPearl improved FBUT by 2.6 s (p < 0.01) and OSDI by 12.6 (p < 0.01). No difference between treatments was detected at 6 months (p = 0.11 for FBUT and p = 0.71 for OSDI), nor were there differences in the other tested parameters between the treatment groups. Blephasteam and TheraPearl are equally effective in treating mild to moderate MGD in a Norwegian population after 6-months of treatment.

Clinicaltrials.gov ID: NCT03318874; Protocol ID: 2014/1983; First registration: 24/10/2017.

## Introduction

Meibomian gland dysfunction (MGD) is the most common cause of dry eye disease (DED) and affects between 5 and 50% of the world’s population^1^. MGD is defined as a chronic, diffuse abnormality of the sebaceous meibomian glands, which commonly is characterized by obstruction of the glandular duct and/or changes in the quality/quantity of glandular secretion^2^. The condition, which is associated with age, sex, hormonal disturbances and environmental factors^3^, leads to tear film alterations and ocular surface disease. Importantly, obstruction of the meibomian gland duct can lead to ductal dilation and subsequent loss of secretory cells in the gland^3^. Consequently, the amount of secretion produced by the gland (meibum) is reduced. The obstruction is most likely also responsible for degeneration of the meibum. In MGD, the amount of branched chain fatty acids and cholesterol in the meibum is increased, which results in a more waxy and viscous secretion^3^. Dysfunction of the meibomian glands could lead to increased evaporation of the tear film and evaporative DED^1^.

The treatment goal of MGD is to improve the function of the meibomian glands by opening occluded gland ducts and improve meibomian gland function^4^. The eyelids maintain about 33 °C under normal ambient room temperature. In healthy subjects, the phase-transition of meibum is about 28 °C (when meibum transitions from an ordered and gel-like phase to a disordered fluid-like phase)^5^. MGD patients have a phase-transition at a higher temperature of about 35 °C^6–9^. This explains the rationale behind applying heat to the eyelids of MGD patients as it brings the meibum in the eyelids above its phase-transition temperature, enabling it to freely flow out of the meibomian glands to comprise the outermost layer of the tear film, thereby reducing evaporation of the aqueous layer^3^.

Application of heat and eyelid massage are considered mainstay treatments for all MGD patients. Several methods exist for heat delivery to the eyelids, including a heated wet towel placed over the eyelids, in-office modalities, steam-based systems, and dry-heat eyelid masks^10–19^. In addition to heat therapy, other treatments, including lubricating eye drops, topical cyclosporine, antibiotics, and manual meibomian probing and expression, should always be considered^10^. Despite heat therapy’s proven efficacy^20–24^, compliance remains a challenge^25^ and it is still unclear whether the type of eyelid heating technique can impact efficacy of the treatment. Accordingly, we sought to investigate whether heat delivered indirectly as steam or directly as dry heat was more effective as a treatment for MGD.

For effective treatment of MGD, temperatures of warm compresses need to exceed 40 °C^26–28^, setting the basis for treatment with heating devices. Several studies have shown that Blephasteam (Spectrum Thea Pharmaceuticals LTD, Macclesfield, UK), a steam-based system, is effective at delivering heat to the eyelids and treating MGD^19,29–31^. Only one other trial has compared Blephasteam to another device^32^.

**Figure 1:**
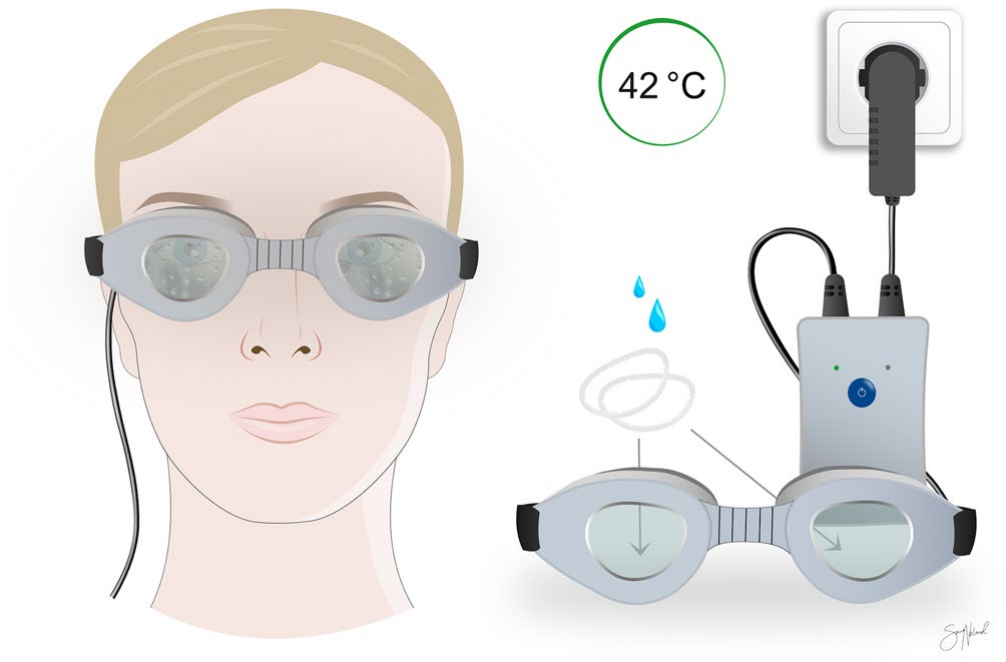
Blephasteam (Spectrum Thea Pharmaceuticals LTD, Macclesfield, UK) warms the eyelids by turning water in cotton rings into steam while placed over the eyes for 10 min. Figure created in Affinity Designer version 1.8 (Serif (Europe) Ltd., Nottingham, UK).

The first study of a steam-based device was tested on healthy subjects and showed its ability to increase the lipid layer thickness and decrease symptoms of ocular discomfort^33^. Blephasteam resembles swimming goggles, making a seal with the periocular skin. A wet cotton ring is placed on the inside of the goggle chambers. After 15 min of preheating, its water evaporates and delivers heated steam, which is about 42° when it reaches the eyelids (Fig. 1).

**Figure 2:**
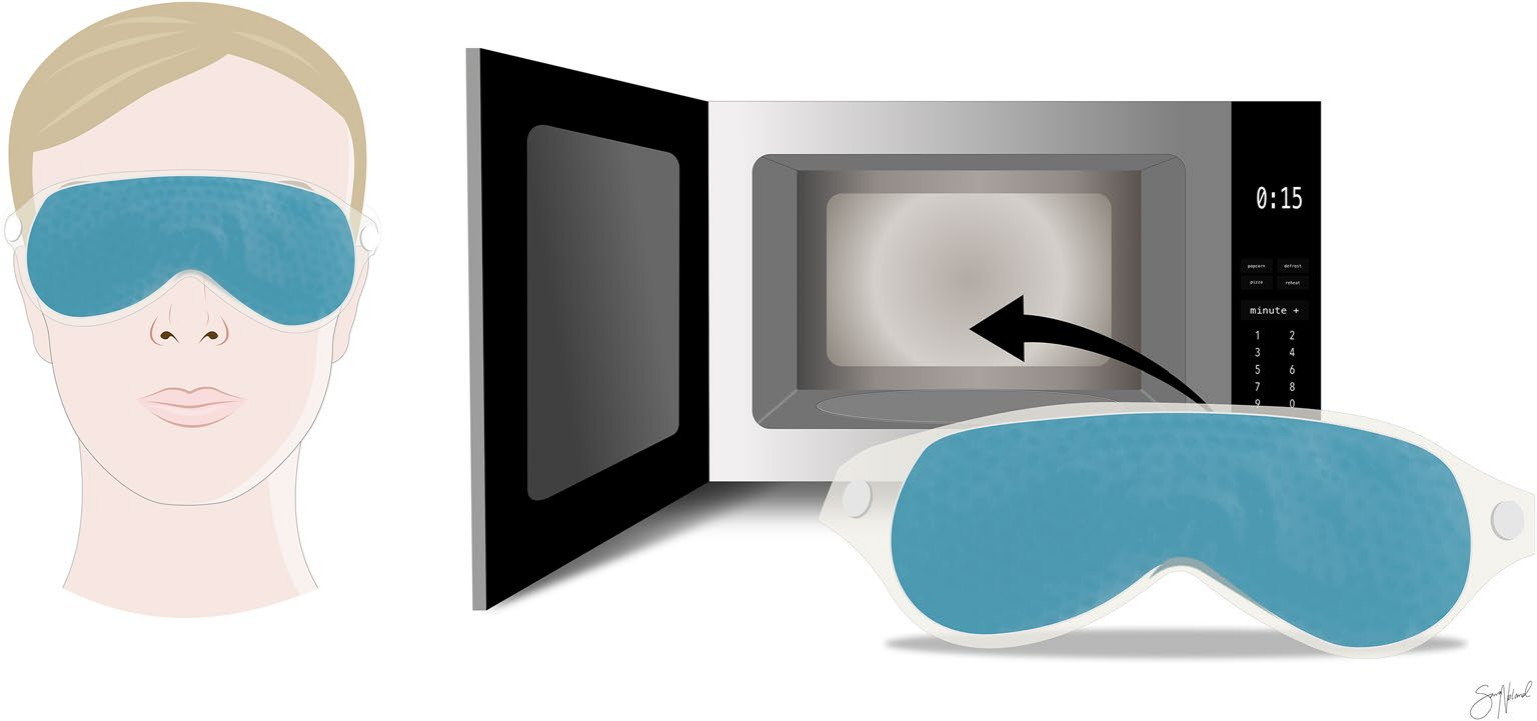
TheraPearl eye mask (Bausch & Lomb Inc., New York, USA) is preheated in a microwave before being placed on the eyelids for about 10–15 min. Figure created in Affinity Designer version 1.8 (Serif (Europe) Ltd., Nottingham, UK).

There are many commercially available eye masks and eyebags that deliver dry heat to the eyelids. TheraPearl Eye Mask (Bausch & Lomb Inc., New York, USA) is a readily available mask in many parts of the world and was chosen as a representative for this group of devices. The mask consists of hundreds of tiny gel beads that are designed to retain heat after being heated in a microwave for 10–15 s before being placed on the patients’ closed eyelids for about 10–15 min (Fig. 2). Only two studies have been conducted specifically on TheraPearl and it has been shown to be efficient at retaining heat^34,35^. Similar eye masks have also been shown to be effective in treating MGD^11,32,36–38^.

Although numerous studies have been conducted on eyelid heating devices, it is still uncertain whether heat delivered as steam or as dry heat is equally effective in treating MGD. Blephasteam has previously been shown to be superior to other modalities and it was therefore hypothesized that it would perform better in this study^32^. In this study, we aimed to compare the effects of the steam-based heat delivery system of Blephasteam with the dry heat delivery mechanism of TheraPearl in a Norwegian population with MGD-related DED.

## Methods

### Ethics

The study was conducted in accordance with the Declaration of Helsinki. The Regional Committee for Medical & Health Research Ethics (REC), Section C, South East Norway, approved the study (reference: 2014/1983). Written informed consent was obtained from all participants.

### Study design and patients

We conducted a 6-month open-label randomized comparative trial, comparing Blephasteam and TheraPearl in patients with MGD-related DED. From 2017 until 2020, eligible patients were recruited from two non-referral clinics: the Norwegian Dry Eye Clinic (Oslo, Norway) and Lavista Eye Clinic (Lillestrøm, Norway). Patients were examined at baseline prior to treatment initiation, and at three and six months after treatment initiation. Towards the end of the recruitment period, the final eight patients that were included had a follow-up of only 3 months. This was done to include as many patients as possible within the duration of the trial, as following these for 6 months was not logistically possible. Patients had to return all treatment apparatus if they did not complete the study, but were otherwise allowed to keep it. No other means of compensation was provided to the patient volunteers.

### Ocular surface work-up

All patients completed a self-report questionnaire at each visit (Ocular Surface Disease Index [OSDI]) to quantify their burden of symptoms during the prior week^39^. OSDI was used as the primary subjective outcome measure. A comprehensive ophthalmological work-up was conducted in the following order: 1. After administration of 5 μl 20 mg/ml sodium fluorescein, fluorescein breakup time (FBUT) was measured in seconds from a blink until the first appearance of a break in the tear film^40,41^, 2. ocular surface staining (OSS) scored with the Oxford grading scheme^42^, 3. Schirmer’s test without anesthesia (mm)^40^, and 4. meibomian quality (MQ)^43^ and meibum expressibility (ME)^43^. FBUT was used as the primary objective outcome measure. All examinations were performed at the same clinic (The Norwegian Dry Eye Clinic) by the principal investigator (JO).

### Inclusion/exclusion criteria

Patients older than 18 years were consecutively invited to participate in the study if they were diagnosed with MGD as defined by the International Workshop on MGD (Table 1)^44^. Patients with glaucoma, ocular allergy, autoimmune disease, contact lens-wear during the study, current punctal plugging, pregnant/lactating, candidate for topical anti-inflammatory therapy, or cicatricial MGD were excluded.

**Table 1:** Diagnostic criteria for meibomian gland dysfunction.

Ocular surface disease index questionnaire	Score > 12
Non-invasive tear film breakup time	< 10 s in at least one eye
Schirmer-1 test	> 5 mm after 5 min in at least one eye
Quality or expressibility score	≤ 20 years old: > 1 > 20 years old: ≥ 1

### Randomization

Patients were randomized to one of two groups. A system of block randomization was used in order to allocate a similar number of participants to each group^45^. Patients agreed to sign a participation agreement before they were told which treatment they were randomized to. Both the patient and the principal investigator (JO) were aware of group allocation as blinding was not logistically possible for this trial.

### Interventions

#### Blephasteam

Patients were instructed to use the goggles in accordance with guidelines from the manufacturer. First the goggles were to be preheated for 15 min before a wet cotton ring was placed inside each chamber. When placed over the eyes, heat was delivered as steam to the eyelids. The device was used once daily with each treatment session lasting 10 min.

#### TheraPearl Eye Mask

Following instructions from the manufacturer, patients heated the mask in a microwave for 10–15 s at 700–1200 Watt, and thereafter placed it upon the eyelids. Patients were instructed to never use the device if the heat caused pain. The mask was used once daily for 10–15 min.

#### Eyelid massage and artificial tears

All patients were instructed in massage of each eyelid for about 10–15 s after each treatment session with either Blephasteam or TheraPearl. They were instructed to use a hyaluronic acid based artificial tear substitute (Hylo-Comod, Ursapharm, Saarbrücken, Germany) as needed to relieve acute symptoms of DED.

### Monitoring of compliance

All patients were asked to use their treatment daily. In order to track compliance, the patients were provided a diary in which they registered the treatment.

### Data management and statistical analysis

For each patient, both left and right eye results were recorded. A descriptive analysis was performed with an independent sample test to compare age and the values at all three visits. A chi-square test was used to determine if there was any difference in the ratio of females to males between the groups. Furthermore, a multivariable logistic regression analysis with all the outcome measures was used to compare the efficacies between the treatments. In order to compare the efficacies of treatment over time, we analyzed two primary variables, namely OSDI and FBUT. Differences in OSDI between visits with Blephasteam and TheraPearl were estimated using generalized linear regression model; FBUT was modeled using a linear mixed effects model to account for inter-eye correlation, with age, sex, visits and treatments considered as fixed effects. Differences among subjects’ eyes were considered as successively nested random effects (i.e., subjects within treatment group and eyes within subject).

We compared patients' compliance under the two different treatments. Compliance, calculated as the number of days per week that the patients were using the treatment, was recorded from initial visit until 6-months follow-up if applicable. We modeled the weekly compliance percentage using a mixed effect model in which age, sex, and the interaction between treatment group and time were considered as a fixed effect. The compliance of each patient could vary randomly in terms of the intercept (starting compliance) and the effect over time. The autocorrelation (the correlation between compliance of the same patients across follow-up period in the data) was also considered in the model using an autoregressive process with a lag of one week. In Fig. 4, fitted smoothed conditional mean compliance using loess method were plotted for each treatment. Statistical analyses of linear and nonlinear mixed effect models were performed with R version 4.0 with package *nlme*, version 3.1-148 (The R Foundation for Statistical Computing, Welthandelsplatz 1, 1020 Vienna, Austria).

## Results

### Characteristics of study subjects at each visit

**Figure 3:**
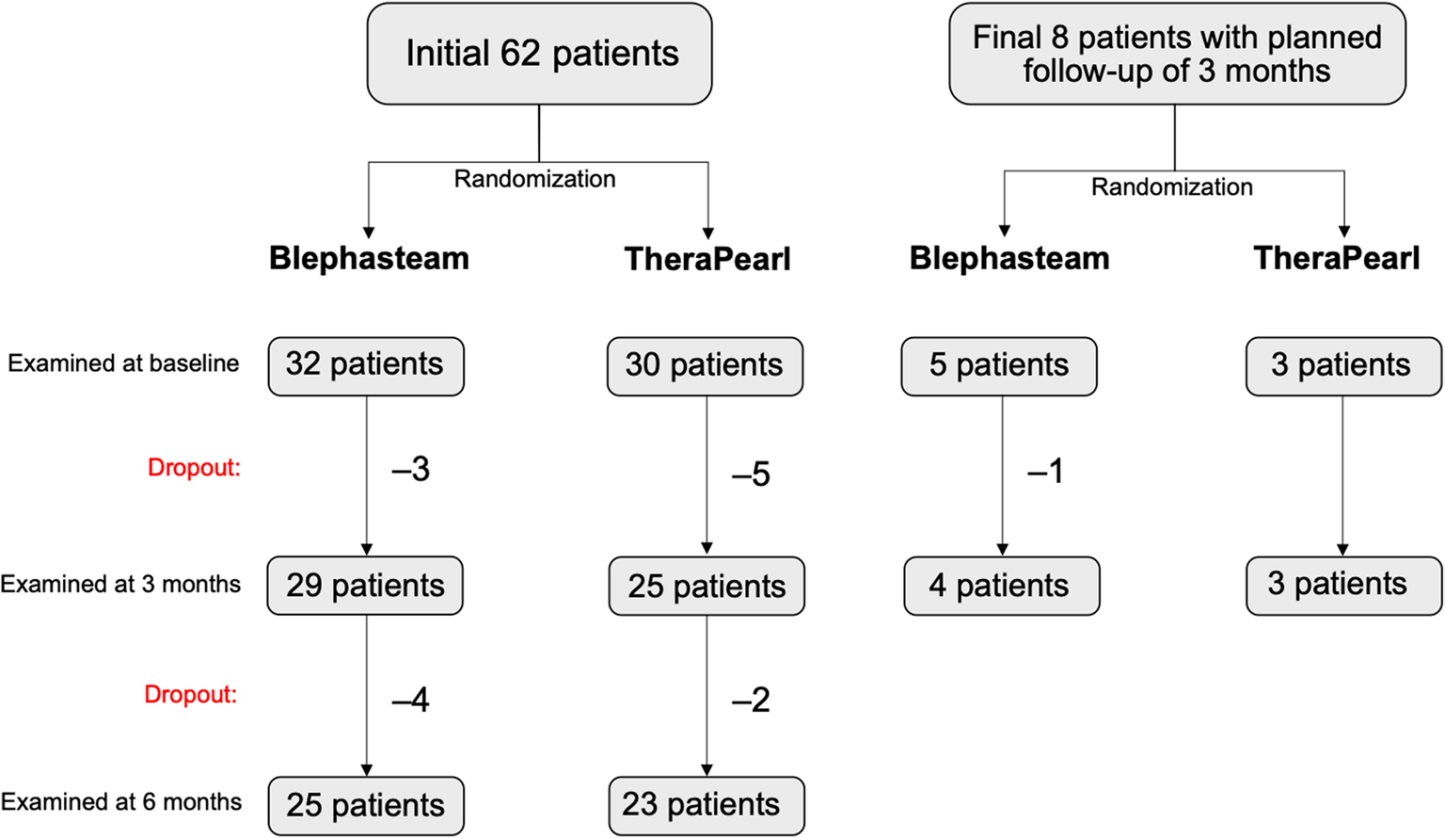
Patient inclusion, treatment allocation, and dropout. Figure created in PowerPoint version 16.53 (Microsoft, Washington, USA).

Seventy patients were enrolled (37 randomized to Blephasteam and 33 to TheraPearl). A flowchart displays the number patients included, treatment allocations, and dropout (Fig. 3). We found no statistical difference between the two groups at baseline with regard to age, sex, or outcome measures, except for OSS at 3 months (Table 2).

**Table 2:** Characteristics of study subjects at baseline, 3, and 6 month visits.

	Baseline			3 months			6 months		
	Blephasteam (n = 37)	TheraPearl (n = 33)	P-value^a^	Blephasteam (n = 34)	TheraPearl (n = 28)	P-value^b^	Blephasteam (n = 25)	TheraPearl (n = 23)	P-value^c^
Mean age	52.2 (46.8, 57.6)	55.1 (49.1, 61.2)	0.46	53.4 (47.8, 59.1)	57.1 (50.8, 63.5)	0.38	52.8 (46.4, 59.2)	58.0 (51.4, 54.6)	0.25
Sex (female)	70.3%	69.7%	0.96	69.7%	67.9%	0.88	62.5%	77.3%	0.28
OSDI	38.7 (31.9, 45.5)	32.9 (26.1, 39.7)	0.23	24.5 (17.1, 31.8)	22.0 (14.2, 29.8)	0.64	23.2 (14.3, 32.2)	21.2 (14.8, 27.6)	0.70
FBUT (s)	4.7 (4.0, 5.4)	4.9 (4.0, 5.8)	0.74	7.2 (5.9, 8.4)	7.3 (5.5, 9.1)	0.88	8.5 (6.6, 10.3)	6.8 (5.2, 8.4)	0.16
OSS^†^	1.50 (1.00, 1.50)	1.00 (1.00, 1.01)	0.58	1.50 (1.49, 2.00)	1.00 (1.00, 1.00)	** < 0.01**	1.50 (1.00, 2.50)	2.00 (1.50, 2.50)	0.17
Schirmer’s test (mm/5 min)	17.7 (14.2, 21.2)	18.9 (14.9, 22.9)	0.66	17.8 (14.0, 21.8)	17.4 (13.2, 21.5)	0.86	22.3 (17.2, 27.5)	17.1 (13.0, 21.2)	0.11
MQ^†^	7.00 (6.50, 8.00)	6.50 (5.50, 7.50)	0.76	5.5 (4.5, 6.0)	5.5 (5.0, 6.5)	0.53	5.50 (4.50, 6.50)	5.00 (4.50, 6.00)	0.40
ME^†^	2.00 (2.00, 2.50)	2.00 (1.50, 2.00)	0.84	1.50 (1.49, 1.50)	1.50 (1.00, 1.50)	0.81	1.00 (1.00, 1.50)	1.50 (1.00, 2.00)	0.34

Values are displayed as means with a 95% confidence interval.

†Median values were reported.

^a^p-value between the two groups at baseline.

^b^At 3 months.

^c^At 6 months. P-values given in bold are considered statistically significant.

*OSDI* ocular surface disease index measured on a scale from 0 to 100, *FBUT* fluorescein breakup time, *OSS* ocular surface staining scored with the Oxford grading scheme from 0 to 15^42^, *MQ* meibomian quality scored on a scale from 0 to 24, *ME* meibomian expressibility scored on a scale from 0 to 3.

### Comparison of efficacy between treatments

We studied if changes in any of the measured parameters after treatment were significantly different between the two treatment groups. Results of the multivariable logistic regression analysis are shown in Table 3. At the three-month follow-up, a significant but minor reduction in OSS was detected in the TheraPearl group, whereas at six months a significant but minor reduction in OSS was noted in the Blephasteam group. There were no significant differences in any of the other outcome measures.

**Table 3:** Results of the analysis comparing efficacy between the treatments.

	Change from baseline to 3 months			Change from baseline to 6 months			Change from 3 to 6 months		
	Blephasteam	TheraPearl	p-value	Blephasteam	TheraPearl	p-value	Blephasteam	TheraPearl	p-value
OSDI	−14.0 (−18.6, −9.4)	−11.1 (−16.2, −6.1)	0.49	−13.7 (−19.4, −7.9)	−12.6 (−18.1, −7.2)	0.71	−0.7 (−5.5, 4.1)	−0.12 (−3.7, 3.5)	0.92
FBUT (s)	2.5 (1.6, 3.4)	2.7 (1.5, 3.9)	0.80	3.9 (2.8, 5.1)	2.6 (1.4, 3.8)	0.11	0.8 (−0.4, 1.9)	0.9 (0.0, 1.7)	0.89
OSS^†^	0 (−0.5, 1)	−1 (−1.5, −1)	0.01**	−1 (−1.5, 0.5)	0 (−1, 1)	0.24	−1 (−1.5, 0)	1.5 (1, 2.5)	< 0.01**
Schirmer’s test (mm/5 min)	0.14 (−2.3, 2.6)	0.14 (−1.5, 1.8)	0.99	0.43 (−1.8, 2.6)	0.72 (−1.2, 2.7)	0.84	0.69 (−1.7, 3.0)	2.0 (0.3, 3.9)	0.36
MQ^†^	−2.5 (−3.5, −1)	−2 (−3, −0.5)	0.47	−3 (−4.5, −1.5)	−2 (−3.5, −0.5)	0.63	−1 (−2, 0)	−1 (−2, 0.5)	0.72
ME^†^	−1.5 (−1.5, −1)	−1 (−1.5, −1)	0.98	−1.5 (−2, −1.5)	−1.5 (−2, −1)	0.29	−1 (−1.5, −1)	−1 (−1, 0)	0.50

Mean changes between time points were reported preceding 95% confidence intervals in parentheses.

†Median changes were reported.

**p < 0.05, the negative significant value indicates TheraPearl decreased OSS by 1 more than Blephasteam and vice versa at 6 months.

*OSDI* ocular surface disease index measured on a scale from 0 to 100, *FBUT* fluorescein breakup time measured in seconds, *OSS* ocular surface staining scored with the Oxford grading scheme from 0 to 15^42^, *MQ* meibomian quality scored on a scale from 0 to 24, *ME* meibomian expressibility scored on a scale from 0 to 3.

### Longitudinal study of differences in FBUT and OSDI

Analysis using the generalized linear mixed model adjusted by age and sex indicated no significant differences in FBUT and OSDI between the two treatment groups regardless of the length of follow-up (Table 4). However, when pooling all patients together for the purpose of analysis, patients improved significantly at three months follow-up relative to baseline in terms of both longer FBUT and lower OSDI. In this study cohort, male patients had a significantly longer FBUT compared to female patients. Males also had a greater increase in FBUT after 3 months of treatment.

**Table 4:** Results of the general linear model corrected for age and sex.

	FBUT (s) (95% CI)	OSDI score (95% CI)
**Between-treatment analysis, comparing Blephasteam to TheraPearl**		
Overall treatment effect^a^	0.30 (−0.99, 1.58)	−0.49 (−7.65, 6.66)
Treatment effect at 3 months compared to baseline	0.28 (−1.05, 1.61)	3.33 (−2.36, 9.02)
Treatment effect at 6 months compared to 3 months	−0.98 (−2.53, 0.57)	−1.53 (−9.37, 4.80)
Treatment effect at 6 months compared to baseline	−0.71 (−2.17, 0.74)	1.79 (−4.44,8.03)
**Pooled analysis of all study patients**		
Age (years)^b^	−0.04** (−0.07, −0.01)	−0.15 (−0.35, 0.03)
Males compared to females^c^	2.43*** (1.04, 3.82)	−4.87 (−12.59, 2.85)
3 months compared to baseline^d^	2.20*** (1.42, 2.97)	−11.30*** (−14.67, −7.92)
6 months compared to 3 months	0.30 (−0.54, 1.15)	0.22 (−3.44, 3.88)
6 months compared to baseline	2.50*** (1.66, 3.34)	−11.07*** (−14.73, −7.41)
Treatment effect at 3 months compared to baseline comparing males to females^e^	1.33* (−0.06, 2.76)	−4.72 (−10.81, 1.39)
Treatment effect at 6 months compared to 3 months comparing males to females	1.32 (−0.26, 2.88)	−3.65 (−11.38, 4.25)
Treatment effect at 6 months compared to baseline comparing males to females	2.68* (1.09, 4.23)	−8.37* (−15.18, −1.55)

FBUT was also corrected for interocular differences between the eyes.

*CI* confidence interval, *FBUT* fluorescein breakup time, *OSDI* ocular surface disease index.

^a^I.e. Overall Blephasteam improved FBUT by 0.30 more than Therapearl (not significantly).

^b^I.e. an increase in age by 1 year decreases in FBUT by 0.038.

^c^I.e. males have an average FBUT of 2.43 s more than females.

^d^I.e. patients improved FBUT by 2.10 s after 3 months of treatment.

^e^I.e. males have an average increase in FBUT by 1.33 s more than females after the first 3 months of treatment.

*p < 0.1; **p < 0.05; ***p < 0.01.

### Compliance

**Figure 4:**
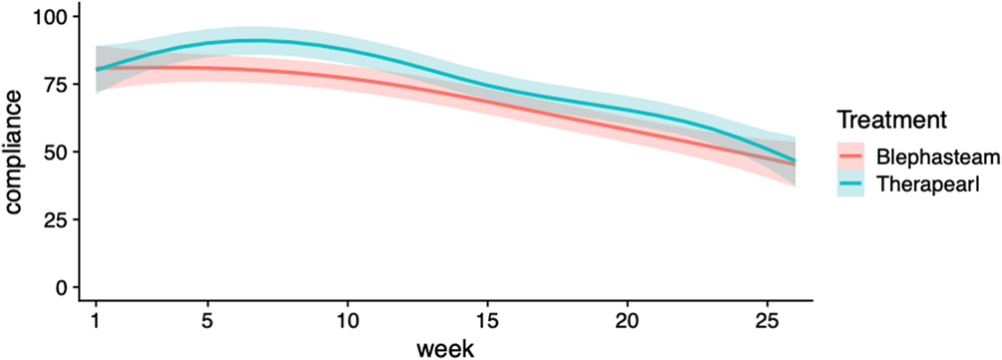
Compliance calculated as the average number of days per week that the patients were using the treatment. Smoothed conditional mean compliance were fitted using loess method for each treatment. 95% confidence bands were shown as shaded ribbon in respective colors. Figure created in R version 4.0 with ggplot2 package, version 3.3.5 (The R Foundation for Statistical Computing, Welthandelsplatz 1, 1020 Vienna, Austria).

We observed a decrease in compliance regardless of treatment group in the study period (Fig. 4). The statistical model confirmed decreasing weekly compliance across treatment groups over the study period ([−0.018, −0.011], p < 1e−5). However, we found no statistical difference in overall compliance comparing the two treatments (p = 0.748), age, or sex.

### Minimal clinically important difference

Table 5 shows the percentages of patients improving by what has been described as minimal clinically important difference^46,47^. This term is defined as “the smallest difference in a score in the domain of interest which patients perceive as beneficial and which would mandate, in the absence of troublesome side effects and excessive cost, a change in the patient’s management”^48^.There were no statistical differences in the proportions between the groups.

**Table 5:** Minimal clinically important difference.

	OSDI decrease by > 10 points		FBUT increase by > 5 s	
	0 to 3 months	0 to 6 months	0 to 3 months	0 to 6 months
Blephasteam	58%	50%	24%	22%
TheraPearl	50%	44%	32%	27%
Between treatment P-value	0.61	0.79	0.68	0.90

*OSDI* ocular surface disease index, *FBUT* fluorescein breakup time.

## Discussion

Both subjective and objective MGD parameters improved three and six months after initiation of treatment with either Blephasteam or TheraPearl. There was no significant difference in efficacy between the treatments after 6 months of treatment.

FBUT is one of the most common tests used to diagnose and monitor DED and was chosen as the primary objective efficacy measure in this study. FBUT improved significantly for both groups from baseline, without any difference detected between the treatments. Additionally, our findings indicate that males generally experienced a greater increase in FBUT after 3 and 6 months of treatment (mean 1.33 s and 2.68 s respectively relative to females), suggesting that treatment may be more efficacious in males. FBUT has some limitations^49–53^. Fluorescein itself reduces the stability of the tear film^54,55^ and the scoring of FBUT is not always exact. To this end, a standardized amount of fluorescein was instilled into each eye using a pipette, and the same observer performed measurements at each visit to eliminate the possibility of inter-observer bias. In addition, all measurements were repeated three times during each visit, with the mean value being recorded.

Ocular surface staining is used to assess corneal epithelial damage and can be caused by a range of ocular diseases, including DED^56^. Our results indicate a slight decrease in OSS in the TheraPearl group during the first three months of treatment, and a slight decrease for Blephasteam in the subsequent three months of treatment. Although the differences were statistically significant between groups, they were not considered clinically important.

There is often great discrepancy between symptoms and signs in DED, making it important to assess subjective measurements in addition to the objective ones^57^. OSDI was chosen as the primary subjective outcome as it is a commonly used and validated questionnaire for DED and MGD^44,46,47^. There was a significant decrease in OSDI after the first three months with a sustained benefit at six months. The differences in FBUT with regards to sex and age were not seen for OSDI-scores.

It is important to consider the minimal clinical difference to detect^48^. It was previously reported that a change of ten points is clinically relevant for OSDI^46^. This change was seen in about half the patients, irrespective of the groups. A change of five seconds was suggested as the minimal clinical difference to detect for FBUT^47^, which was seen in about a quarter of the patients, also without any statistical difference between the groups. An increase in 5 s in FBUT is proportionately much greater than a decrease in ten points for OSDI. With the baseline average of about five seconds, a 100% increase in FBUT is required to reach ten seconds, whereas a similar improvement in OSDI by ten points only requires a change of about 30%, which may explain the discrepancy between the improvements by minimal clinical difference between FBUT and OSDI. The proportion of improvement by the minimal clinical difference was higher after 3 months than at 6 months. This may be due to falling compliance after 3 months.

At the time of writing, six published randomized controlled studies were conducted comparing Blephasteam to other treatments^16,22,32,33,38,58^. These had shorter follow-up, of 2^22^, 6^19^, and 12 weeks^32^, and three trials evaluated the outcome parameters immediately after application of the device^16,33,58^. We chose to follow our patients for six months, the longest follow-up period of any randomized trial to date, to see if prolonged usage had any additional advantage. We found no statistical improvement from 3 to 6 months, indicating that the greatest improvement in parameters comes in the initial three months of treatment.

In 2014, Sims et al. reported a comparison between Blephasteam, EyeGiene (in principle similar to TheraPearl), and a hot towel in a Chinese population^32^. As the prevalence and severity of dry eye is higher in Asian populations^1,59^, it was uncertain if the results would differ in a Nordic population. The earlier results indicated that Blephasteam was more effective at relieving symptoms than EyeGiene and hot towels, with the latter two treatments being equally effective^32^. The authors suggested that the difference in efficacy may be due to lower compliance in the non-Blephasteam groups due to technical difficulties with the treatments as well as lower symptom severity at baseline. In the current study we found no difference in symptom severity at baseline, and no patients reported any technical difficulties using either device, without any difference in compliance detected between the groups.

Patients in this study were given an artificial tear substitute (Hylo-Comod), which in itself could have decreased symptoms and signs^25^. Many of our patients, however, were already using artificial tear substitutes before being recruited and its effect of the endpoints was therefore considered to be minimal. Moreover, the use of artificial tear substitutes resembles the real-world situation where patients would likely use tear substitutes in combination with other therapies. In addition, it was considered unethical to discontinue such treatment for the duration of the study. We therefore provided the same artificial tears to all patients.

A strength of the current study is the large sample size of patients that all require first-line therapies for MGD. Patients were recruited from both a non-referral ophthalmology clinic (The Norwegian Dry Eye Clinic) and a non-referral optometry clinic (Lavista) to decrease selection bias from a specialized dry eye clinic. This adds to the diversity in the severity of DED and thereby increases the generalizability of the study. Other strengths of our study include the design (prospective, randomized) and by far the longest follow-up period reported to date. As steam-based heating methods are considerably more expensive both in terms of purchase and maintenance (Blephasteam rings, Spectrum Thea Pharmaceuticals Ltd, Macclesfield, UK) than dry heat methods, the results of our study could also have economic implications for the patients.

Inclusion was limited to patients only requiring eyelid heating therapy and artificial tears. Those with a high degree of ocular surface staining at baseline were thus excluded as those subjects would require anti-inflammatory treatment^25^. This may have led to a selection bias as more severe presentations of DED were not included in the study. Further studies are needed to assess these treatments in more severe DED. Other limitations of our study include that patients and examiners were not blinded, in addition to a relatively high drop-out rate and declining compliance. Fortunately, the highest drop in compliance was beyond the second follow-up visit at 12 weeks, which may reflect the success of the treatments as patients did not feel the need to continue with daily treatments.

As DED is one of the most common reasons for seeking ocular medical attention, it is important for ophthalmologists, general practitioners, optometrists, and opticians to have readily available, effective, and convenient treatments to recommend^60^. It is well established that treatment compliance in MGD-related DED is a major obstacle as the treatment is time-consuming and needs to be repeated every day for weeks to years^25^. Well documented treatments will motivate the patients and increase compliance.

Patients can also be motivated by the knowledge that treatment can result in improvement in as little as three months. As the results of the current study are contrary to our hypothesis, based on other studies showing Blephasteam to be superior to simple eye lid warming devices^32^, further studies are warranted to confirm our results. The reason for non-superiority in the present study could be due to our choice of product as TheraPearl has previously never been compared to Blephasteam, as well as the severe cases of MGD that require anti-inflammatory treatment were excluded. Another reason could be related to where the study was performed. Potential benefits of steam may not be applicable in certain geographic areas.

Our study provides further documentation of the favorable effects of applying heat to the eyelids and, importantly, to what extent this therapy can improve symptoms and signs of MGD. We conclude that Blephasteam and TheraPearl are equally effective in treating MGD in the long term, at least in a southern Norwegian population with mild to moderate MGD. Considering the vast difference in price between the two treatment options (Prices based on the Norwegian website apotekhjem.no accessed January 2021: 14 USD for TheraPearl and 210 USD for Blephasteam^61,62^), a simple heating system, such as TheraPearl, seems to be a good choice. With an estimate of more than a billion people having MGD in the world^63^, the results of our study have important implications for accessibility, as it shows that the more economical choice is just as effective.
